# Understanding avian egg cuticle formation in the oviduct: a study of its origin and deposition^†^

**DOI:** 10.1093/biolre/iox070

**Published:** 2017-07-04

**Authors:** Peter W. Wilson, Ceara S. Suther, Maureen M. Bain, Wiebke Icken, Anita Jones, Fiona Quinlan-Pluck, Victor Olori, Joël Gautron, Ian C. Dunn

**Affiliations:** 1 The Roslin Institute, University of Edinburgh, Easter Bush Campus, Midlothian, Scotland, UK; 2 College of Medical, Veterinary and Life Sciences, Institute of Biodiversity, Animal Health and Comparative Medicine, University of Glasgow, Glasgow, Scotland, UK; 3 Lohmann Tierzucht, Cuxhaven, Germany; 4 School of Chemistry, University of Edinburgh, Joseph Black Building, Edinburgh, Scotland, UK; 5 Aviagen, Midlothian, Scotland, UK; 6 INRA, UR83 Recherches Avicoles, Nouzilly, France

**Keywords:** oviduct, ovum, uterus, vasopressin, gonadotropin-releasing hormone

## Abstract

The cuticle is a unique invisible oviduct secretion that protects avian eggs from bacterial penetration through gas exchange pores. Despite its importance, experimental evidence is lacking for where, when, and what is responsible for its deposition. By using knowledge about the ovulatory cycle and oviposition, we have manipulated cuticle deposition to obtain evidence on these key points. Cuticle deposition was measured using staining and spectrophotometry. Experimental evidence supports the location of cuticle deposition to be the shell gland pouch (uterus), not the vagina, and the time of deposition to be within the final hour before oviposition. Oviposition induced by arginine vasotocin or prostaglandin, the penultimate and ultimate factors for the induction of oviposition, produces an egg with no cuticle; therefore, these factors are not responsible for cuticle secretion. Conversely, oviposition induced by GNRH, which mimics the normal events of ovulation and oviposition, results in a normal cuticle. There is no evidence that cuticle deposition differs at the end of a clutch and, therefore, there is no evidence that the ovulatory surge of progesterone affects cuticle deposition. Overall, the results demonstrate that the cuticle is a specific secretion and is not merely an extension of the organic matrix of the shell. Cuticle deposition was found to be reduced by an environmental stressor, and there is no codependence of the deposition of pigment and cuticle. Defining the basic facts surrounding cuticle deposition will help reduce contamination of hen's eggs and increase understanding of the strategies birds use to protect their eggs.

## Summary Sentence

The cuticle is an invisible but important defense against microbes entering avian eggs. Where and when in the oviduct it is deposited on the egg and what influences its deposition have been demonstrated in the paper.

## Introduction

The cuticle is an invisible layer deposited on the outside of avian eggs [1], filling the gas exchange pores and preventing bacterial contamination [2, 3]. However, despite the knowledge of its existence and its function for at least 130 years [4, 5], there remains confusion about its formation, its relationship with protoporphyrin pigmentation of eggs and indeed where it is formed; similar questions were posed 80 years ago [6].

The cuticle can be found on eggs from many avian species [7], with its thickness greater on eggs laid in nests with a greater microbial challenge, particularly those of aquatic birds [7, 8]. The presence of cuticular mineralized nanospheres has been suggested to be correlated with the wetness and warmness of the nest [9], and the reports of eggs with no cuticle are from species such as *Melopsittacus undulates (budgerigar)*, which are from drier environments, and overall there is a relationship between damp environments and cuticle occurrence [9, 10]. Although separated, egg and feces exit through the avian cloaca, which along with dirty nest sites provides opportunities for egg contamination [11]. Having an aqueous and antimicrobial barrier, such as the cuticle, would seem to be an appropriate evolutionary response to these challenges. We demonstrated that the quantity of cuticle was a heritable trait in chickens and, within its normal range of variation, had a significant effect on bacterial penetration of eggs [2]. Complete removal of the cuticle increases both particle and water penetration [12, 13]. A good cuticle will therefore inhibit vertical and horizontal transmission [14, 15] of bacteria that may threaten the viability of the developing embryo.

The cuticle comprises proteins, which we [2] and others [16–18] have identified, in chickens, to be principally Bactericidal/Permeability-Increasing protein (BPI) fold-containing family B member 3 BPIFB3 (ovocalyxin-36), kunitz-like protease inhibitor, matrix extracellular phosphoglycoprotein MEPE (ovocleidin-116)**, ovocleidin-17 OC-17, ovocalyxin 25, clusterin CLU, and retinoic acid receptor responder 1 RARRES1 (ovocalyxin-32). These proteins are also major components of the organic matrix of the eggshell [19]. The cuticle contains polysaccharides related to the glycosylation of the proteins as well as lipids [6, 17, 20], indeed most of the proteins are thought to be heavily glycosylated [21].The antimicrobial activity of these proteins has been hypothesized [18, 22], therefore it is likely that the cuticle acts both as a physical barrier and as a chemically active antimicrobial layer [16, 23]

Where the cuticle is formed appears to be contentious. There are online articles on avian reproduction stating that it is formed in the vagina but proof seems limited for the assertions made. The vagina of birds is a muscular region where the egg spends a short time while it is ejected during oviposition, [24] but it does contain secretory cells and appears to produce antimicrobial factors [25, 26]. The shell gland pouch (SGP) or uterus, by contrast, is where the egg spends the majority of time during egg formation, while the eggshell matrix and the Ca^++^ and CO_3_^−^ ions for mineralization of the matrix are secreted [27]. The evidence is best for the formation of cuticle in the shell gland. It was reported that dermatan sulfate, a strongly sulfated acidic glycoprotein, is present in the epithelial and tubular glands of the shell gland in large amounts towards the end of shell formation [28], but was largely gone after oviposition. In quail, a 32-kD protein, present both on the surface of the egg and in the ciliated cells of the luminal epithelium of the shell gland, accumulates towards the end of egg formation and was largely gone after oviposition [29]. Although the correct size, RARRES1, which is known to be abundant in the outer layers of the shell, especially the cuticle [18], was not thought by Rahman to be the protein they observed. Unfortunately, none of these authors reported on the vagina, so it is not possible to rule it out as a source of the cuticle. However, a study where eggs were removed from the oviduct at different times during formation concluded that the cuticle was formed in the uterus [6].

Another unanswered question is when is the cuticle deposited? Logic might dictate that the cuticle is deposited towards the end of egg formation, as it covers the outside of the egg. The study of Fernandez [28], based on the disappearance of dermatan sulfate from the epithelia of the shell gland, was made 18 h after the previous oviposition; however, it would be a further 6 h until the egg in the shell gland was oviposited. Similarly, Rahman's [29] observations on a protein potentially involved in cuticle formation were made approximately 4 h before the expected time of the next oviposition, so neither study narrowed down the time of cuticle deposition. While it may be supposed that the cuticle is a specific deposition, unrelated to that of the organic matrix of the eggshell, this has not been tested and the similarity of the proteins in the cuticle and in the matrix [2] do not preclude the possibility that the cuticle is in some way a continuation of the process of matrix secretion. Indeed this very question was posed in an early study [6].

It is frequently stated that in chickens the cuticle is related to the deposition of, or contains to some extent, the eggshell pigment in brown eggs [30]. This is supported by observations [17, 31], but there was no evidence for a genetic correlation between the amount of pigment and the amount of cuticle on an egg [2].

Finally, although we know that genetics contributes to variation in the amount of cuticle deposited on an egg [2], the influence of environmental factors on its variation has not been investigated. In an effort to answer the questions posed in the introduction and to move on from Hutt's statement that “the cuticle is part of the shell concerning the formation of which little is known” [32], we have undertaken a series of experiments, most of which utilize the events controlling the ovulatory cycle and subsequent oviposition, to understand the process of deposition of the cuticle.

## Materials and methods

### Animals

Throughout these experiments, commercial Lohmann Brown (Lohmann GB, Worcester, England) layer hens (*Gallus gallus domesticus*) were used, typically weighing 1.8 kg. They were reared on the floor, to peak-of-lay (22–28 weeks), following commercial management practice, except for the lighting, which was 14L:10D. After hens reached peak-of-lay, they were transferred to cages on a 14L:10D lighting regime for experiments 1 and 2, or onto a 28-h ahemeral light/dark cycle (14L: 14D) for all other experiments (except experiment 8) to enable accurate synchronization of ovulation/oviposition times [33]. By maintaining hens on a 28-h (14L:14D) cycle, the ovulatory surge and oviposition can be predicted with great accuracy, as pause days are eliminated, and there is no drift in oviposition time. On a 28-h light/dark cycle, ovulation and oviposition occurs 8 h after dusk [34].

In experiment 8, comparing eggs oviposited by hens that did or did not experience a progesterone surge, hens were maintained in cages on 15L:9D. Hens ovulate and oviposit approximately once every 24 h, but an ovarian follicle takes slightly longer than 24 h to mature [35]. Therefore, hens occasionally have a “pause day,” when no oviposition takes place because no ovulation occurred ∼24 h previously. This is because the follicle is not sufficiently mature to sustain the progesterone feedback required to propagate an ovulatory surge [36]. However, the egg that was in the shell gland when an ovulation did not occur is oviposited [37]. This offers a contrast between eggs which, when oviposited, had or had not experienced the milieu of an ovulatory progesterone surge while in the shell gland, which has been hypothesized as a factor influencing cuticle secretion and deposition [38].

### Administered substances

A number of hormonal factors can induce premature ovulation and/or oviposition; this can be used to study cuticle formation by intervening in the neuroendocrine pathway between brain and ovary.

An injection of chicken gonadotropin-releasing hormone 1 (GNRH1) 10 h before normal oviposition initiates a normal endocrine cascade of hormones including a progesterone surge [39, 40], which results in a premature ovulation and a subsequent ovposition that are temporally linked [41]. Prostaglandins released from the mature follicles during ovulation are believed to initiate the linked oviposition [41]. The administration of prostaglandin, which is most proximate to the stimulation of muscular contractions, leads almost immediately to oviposition [42, 43] but has no effect on ovulation.

Arginine vasotocin (AVT) from the neurohypophysis initiates a premature oviposition by mimicking the final part of the endocrine cascade, resulting in oviposition, stimulating local release of prostaglandin that causes bearing down, uterine contraction, and vaginal relaxation [44–46]. Indomethacin, a nonsteroidal anti-inflammatory drug, blocks prostaglandin synthesis and is known to prevent the action of AVT on oviposition [47]. In the experiments described in this paper, therefore, the following substances were administered in different combinations: indomethacin (I7378, Sigma-Aldrich Company Ltd, Dorset, England) i.m. at a dose of 3.6 × 10^−3^ mol/kg body weight suspended in peanut oil (0.112 mol/m^3^); AVT (ab-142 562, Abcam, Cambridge, England) i.v. at 1.05 × 10^−6^ mol/kg body weight in PBS (initially dissolved in 25% v/v acetic acid to 4.76 × 10^−6^ mol/m^3^ then diluted to 3.8 × 10^−9^ mol/m^3^ with PBS for injection); chicken GNRHI (H-3106, Bachem, St. Helens, England) i.v. at a dose of 28.9 × 10^−6^ mol/kg body weight in PBS (17.3 × 10^−6^ mol/m^3^); the prostaglandin associated with events surrounding oviposition is PGF2α (16 020, Cambridge Bioscience, Cambridge, England) and was given i.v. at a dose of 1.06 × 10^−6^ mol/kg body weight in PBS (33.8 × 10^−9^ mol/m^3^) [48]. Additionally, tamoxifen (CAY-11 629, Cambridge Bioscience, Cambridge, England), which is an estrogen antagonist, was administered at a dose designed to cause mild reduction in steroid tone and therefore oviduct mass and function [49]. We predicted that this would cause a reduction in the quality of the cuticle, and it was administered i.m. at a dose of 37 × 10^−3^ mol/kg body weight suspended in propylene glycol (40.4 × 10^−6^ mol/m^3^).

### Experimental protocols

There are eight experiments described; to make these easier to follow, there is a summary in Table 1.

**Table 1.** Summary of experiments, hypothesis tested, and variables manipulated.

Experiment title	Hypothesis tested	Variable manipulated or substance administered
1: Pen-to-cage transfer to perturb oviduct function.	That stress would reduce cuticle deposition.	Pen to cage transfer
2: Administration of the estrogen-antagonist, tamoxifen, to perturb oviduct function.	That a reduction in steroid tone would reduce cuticle deposition.	Tamoxifen i.m. administration
3: Administration of AVT and indomethacin to influence oviposition time.	That premature oviposition mediated by prostaglandin would result in an egg with normal cuticle.	AVT i.v. and indomethacin i.m. administration 3hrs before an expected oviposition.
4: Administration of AVT to induce premature oviposition at different times in advance of a predicted oviposition.	That cuticle deposition occurred immediately prior to oviposition.	AVT i.v. administration at 1, 3 and 5 hrs before an expected oviposition.
5: Administration of GNRH1 or AVT to induce premature oviposition.	That cuticle deposition after a premature oviposition following a premature ovulation would differ from a premature oviposition unaccompanied by a premature ovulation.	AVT or GnRH i.v. administration 4 and 10 hrs before an expected oviposition respectively.
6: The effect of administration of prostaglandin on cuticle deposition.	That premature oviposition induced by AVT and prostaglandin would be identical.	AVT and prostaglandin i.v. administration 2 hrs before an expected oviposition.
7: The effect of the depigmentation of eggs by nicarbazin on cuticle deposition.	That inhibition of pigment deposition would have no correlated effect on cuticle deposition.	Nicarbazin in feed administration.
8: The effect of a pause day on cuticle deposition.	That the absence of a preovulatory progesterone surge would reduce cuticle deposition.	Examination of eggs from hens experiencing a day with no ovulation.

To understand the external factors that influence the formation of the cuticle, we made subtle changes to the physiology of the laying hen to determine the effect of perturbation to oviduct trophic support on egg cuticle deposition: experiments 1 and 2, described below.

#### Experiment 1: pen-to-cage transfer to perturb oviduct function

Moving hens between environments is a mild stressor that can cause significant regression of the oviduct within 4 days of movement [50]. Hens were housed in 4 floor pens, 6 hens per pen (n = 24), prior to commencement of lay. At peak-of-lay, a pen of hens was transferred to cages in the same room on day 1, day 3, day 7, and day 10 (batch 1–4). Eggs were collected from the floor-pen nest boxes for 2 days prior to transfer (control) and from the cages for 2 days post transfer. A one-way analysis of variance (ANOVA) was performed to analyze the data. The nuisance factor of batch was fitted in the model.

#### Experiment 2: administration of the oestrogen antagonist tamoxifen to perturb oviduct function

The hens described in experiment 1 were acclimatized for 2 weeks in the individual cages. Each hen was treated on two successive days with tamoxifen or vehicle control (n = 11 per treatment). After 1 week, the reciprocal treatment was performed in a Latin square design, so hens acted as their own controls. Eggs were collected on the day prior to treatment (as a control) and on the day of treatment. Data were analyzed as a Latin square design ANOVA, with hen and occasion as row and column. The nuisance factor of batch was fitted in the model.

A further set of experiments examined how inducing premature oviposition, by altering the timing of hormonal signals on hens kept on 28 h ahemeral lighting cycles, influenced cuticle formation: experiments 3–5, described below.

#### Experiment 3: administration of arginine vasotocin and indomethacin to influence oviposition time

If prostaglandins are involved in the deposition of the cuticle, we would expect that manipulating prostaglandin levels would have an effect. Hens (n = 24) were transferred to cages in rooms on 14L:14D cycles in four batches (six hens per batch). Once hens were acclimatized to the cages, they were treated over 4 weeks, with each hen acting as its own control. Once per week, each hen received one of the following treatments in a Latin square design: AVT only; indomethacin + AVT; indomethacin only; vehicle control. Indomethacin was administered 3 h before the AVT injection, which, although at a lower efficiency than AVT alone, was reported to still induce oviposition, but without an increase in prostaglandin [44]. Vehicle injections were given in place of indomethacin and AVT, where appropriate. Eggs were collected on the day prior to treatment (as a control) and on the day of treatment. Data were analyzed as a Latin square design ANOVA, with hen and occasion as row and column. The nuisance factor of room was fitted in the model. The same analyses were used for experiments 4–7.

#### Experiment 4: administration of arginine vasotocin to induce premature oviposition at different times, in advance of a predicted oviposition.

To establish when the cuticle is deposited relative to oviposition, AVT was administered 5, 3, and 1 h prior to the predicted oviposition on a 28-h ahemeral light cycle. Hens (n = 12) were housed in three rooms and assigned treatments in a Latin square design over 3 weeks, each hen receiving all treatments over successive weeks. Eggs were collected on the day prior to treatment (as a control) and on the day of treatment.

#### Experiment 5: administration of GNRH1 and arginine vasotocin to induce premature oviposition

GNRH1 was used to induce a premature oviposition; AVT was administered to result in an oviposition that would match the premature GNRH1-induced oviposition. This allowed a comparison of cuticles from eggs oviposited at the same time, 4–4.5 h premature, but which had experienced a different endocrine milieu before oviposition. Each hen (n = 12) received GNRH1, AVT, or vehicle control in a Latin square design with each hen acting as its own control. Eggs were collected the day prior to (as a control) and the day of treatment.

The previous series of experiments was designed to evaluate the effects of oviposition time and changes to that induced by GNRH1 and AVT on cuticle deposition on eggs. In the following experiment, we wanted to investigate a factor, not directly tested in the previous experiments, that was a candidate for inducing cuticle deposition, prostaglandin [43]. Our aim was to test if PGF2α was directly involved in the secretion and deposition of the cuticle on eggs.

#### Experiment 6: the effect of administration of prostaglandin on cuticle deposition

AVT or PGF2α was given 2 h prior to expected oviposition. Hens (n = 16) were treated on two separate weeks in a Latin square design. Eggs were collected on the day prior to treatment (as a control) and on the day of treatment.

Nicarbazin has been used since the 1950s for the prevention and control of coccidiosis caused by *Eimeria spp.* in poultry [51]. A known side effect is the rapid depigmentation of eggs in brown egg layers [51], probably through the inhibition of expression of the rate-limiting step in protopophyrin production, 5-aminolevulinate synthase (ALAS1) [52]. In the following experiment, we used this effect to investigate whether the amount of pigment (color) and cuticle on the egg surface are linked.

#### Experiment 7: the effect of the depigmentation of eggs by nicarbazin on cuticle deposition

Hens (n = 12) were kept in cages in two rooms for 2 weeks before the start of treatments to allow acclimatization and to gather baseline data. All eggs were collected. The diet was changed to contain 2.35 × 10^−4^ mol/kg of nicarbazin (N3905, Scientific Laboratory supplies, Newhouse, Scotland) (n = 6) or continued with no nicarbazin control (n = 6), balanced across rooms. Eggs were collected for 7 days; the treatments were then reversed, with egg collection for a further 7 days. Eggs from the seventh day of each treatment were measured for comparison.

In the final experiment, we utilized the hens “pause day,” when no oviposition takes place, to enable comparison of eggs that, when oviposited, had or had not experienced the milieu of a progesterone ovulatory surge while in the shell gland.

#### Experiment 8: the effect of a pause day on cuticle deposition

By careful monitoring of an individually housed flock of hens over 3 days, it was possible to characterize 21 hens experiencing a pause day. Eggs oviposited on the day prior to the pause day were compared with eggs from the same hen oviposited on the preceding day. Essentially, we chose hens that had not oviposited on the third day, assuming that the egg previous to that had been oviposited on a day with no ovulation. By using a flock that was at the end of production (∼70 weeks of age), we were able to confirm our assumption by killing the hen and checking for an absence of internal ovulation and a full ovarian hierarchy of follicles. The egg from before the pause day and the one prior to that was measured for cuticle coverage. The hypothesis proposed that we should have a poorer cuticle on eggs oviposited immediately prior to a pause day as there was no progesterone surge. A paired “t” test was used to analyze if there was any difference between the two eggs from the same hen.

### Data analysis and presentation

All analyses in the paper with the exception of the immunohistochemistry were performed using Genstat (13th edition, VSN International Ltd, Hemel Hempstead, England). Details of analysis for each experiment have been given in the relevant experimental methods. Sample sizes were derived from initial power calculations based on observations of variance from previous measurements of the cuticle and projected treatment effects. This was subsequently revised down in light of the size of the effects observed. Animals were assigned to experimental treatments, within rooms where appropriate, by ranking and randomization on body weight.

Data from experiments 1 and 8 are presented as mean and standard error of the mean ± s.e.m. of the observed data from eggs in the treatment or observed groups. Data from experiments 2–7 are presented as mean ± s.e.m. of the difference between the egg before and the egg after treatment from the same hen.

### Shell cuticle and pigment measurement

Cuticle and pigment were measured spectrophotometrically, essentially as described previously [2]. Briefly, reflectance at 640 nm was measured in situ on the eggshell using a USB4000-VIS-NIR spectrometer coupled to an ISP-REF integrating sphere and data collected using Oceanview spectroscopy software (Ocean Optics, Oxford, England). The measured reflectance values were converted to absorbance, which is linearly related to the concentration of the absorbing species. This initial measurement gave a value attributable to protoporphyrin IX pigment. Eggs were then stained with a cuticle-specific dye, which shows no staining when the cuticle is removed and has been used previously [13, 53]. The dye consists of tartrazine/lissamine green B (Sigma-Aldrich, Poole, Dorset, England); eggs are dipped for 30 s then rinsed in H_2_O and dried. Stained eggs were measured again, as above, and the difference between the prestain and poststain absorbance at 640 nm gave a measure of the cuticle deposition. Using these spectrometric measurements, the following values were calculated for the appropriate experiments.

Abs @ 640 nm (pigment): measurement of absorbance at 640 nm on the unstained egg. This value is directly related to the intensity of the pigmentation, or in other words the brownness of the egg.

ΔAbs @640 nm (Δ pigment): difference in absorbance at 640 nm of unstained eggs from the same hen before and after the hen was treated. This indicates the effect of the treatment on the amount of pigment (brownness).

Cuticle ΔAbs @640 nm: difference in absorbance at 640 nm between the unstained egg and the same egg stained with cuticle dye. This is the absorbance attributable to the dye bound to the cuticle and therefore is indicative of amount of cuticle deposition.

Cuticle ΔΔAbs @640 nm: difference between Cuticle ΔAbs @640 nm values for an egg before and after treatment of the hen. This indicates the effect of treatment on the amount of cuticle deposited.

### Measurement of other egg parameters

Standard measurements of egg parameters were also made in some experiments such as weight, and particularly shell thickness on shells without membrane, using a digital micrometer, model 705-1279, with a spherical anvil to account for shell curvature (RS Components Ltd, Corby, England). These were used to calculate before and after treatment the hen received; Δ egg weight (g) and Δ shell thickness (μm).

### Immunohistochemistry

Tissue samples for immunohistochemistry were collected using the same paradigm as experiment 4, except hens were euthanized immediately after the premature oviposition induced by GNRH1 or AVT. This mimicked, respectively, the whole or the final stages of the endocrine cascade leading to oviposition. Tissue samples from the SGP and vagina were immediately fixed in 10% buffered neutral formalin for 24 h. Immunohistochemistry for RARRES1 (commonly known as Ovocalyxin 32) used an antibody previously characterized (Supplementary Table 1) using the protocol described previously [18]. We knew from previous proteomic experiments and from immunohistochemistry that RARRES1 was a major component of the cuticle [2, 18, 54].

Differences to the previous protocol were that rabbit anti-RARRES1 antiserum at 1:50 000 was used in conjunction with a goat antirabbit biotinylated second antibody (E0432, DAKO, Supplementary Table 1) as per the manufacturer's instructions. Binding was with a VECTASTAIN Elite ABC-HRP reagent (Vector Laboratories, Peterborough, England) followed by color development with 3,3-diaminobenzidine tetrahydrochloride chromogenic substrate (K3468, Liquid DAB+ kit, DAKO Denmark A/S, Glostrup, Denmark). Brown staining indicated a positive result for RARRES1. Controls were performed with no primary antibody or with normal rabbit serum substituted for the primary antibody. ImageJ software was used to analyze four images per section by first defining a region of interest (ROI), splitting the color channels, making the ROI binary, and then measuring the percentage of “black” that corresponded to the ICC positively stained regions. The ROI in each case incorporated as many whole epithelial cells as possible on a single image. The % mean area of uterus or vaginal tissue staining positive for RARRES1 was compared using ANOVA (Minitab version 17, Coventry, England).

Ethical statement: experiments were carried out under the Animals (Scientific Procedures) Act 1986, project license 70/7909, and individual experiments were approved by the Institute Ethics Committee.

## Results

### Experiment 1: pen-to-cage transfer to perturb oviduct function

Pen-to-cage transfer resulted in a reduction in the amount of cuticle (Cuticle ΔAbs @640 nm) on eggs laid after the transfer (Table 2). There was no effect of the transfer of hens from pens to cages on pigment or on any other measured egg parameter.

**Table 2.** Effect of pen to cage transfer on cuticle deposition, pigment deposition, egg weight, and shape (n = 24).

	Before transfer^1^	After transfer^1^	ANOVA
Egg parameter	± s.e.m.	± s.e.m.	*P-value*
Cuticle ΔAbs @640 nm	0.529 ± 0.018	0.465 ± 0.017	0.01
Abs @640 nm (pigment)	0.376 ± 0.006	0.384 ± 0.007	0.429
Egg weight (g)	59.0 ± 0.7	59.3 ± 0.8	0.832
Egg length (mm)	55.8 ± 0.3	56.4 ± 0.3	0.176
Egg width (mm)	43.1 ± 0.2	43.5 ± 0.2	0.183

^1^In this experiment values are shown as the mean ± s.e.m. of measurements derived from 2 eggs before and 2 eggs after transfer*.*

### Experiment 2: administration of the estrogen antagonist, tamoxifen, to perturb oviduct function

Tamoxifen, administered to cause a mild reduction in steroid tone, had a small effect on the deposition of cuticle on the egg compared to the control (Table 3). Tamoxifen administration resulted in a small reduction in the amount of pigment on the treated hens’ eggs (ΔAbs @640 nm), but this did not reach significance (Table 3). The change in cuticle deposition due to the treatment (ΔAbs @640 nm) was very small, compared to the changes observed in other experiments in this study and, in fact, the amount of cuticle was reduced after treatment in the control but was unaffected in tamoxifen-treated hens (Table 3). The experimental procedure itself appeared to have an effect on the cuticle; in the control injection group, there was a reduction in cuticle deposition when comparing before and after treatment by a paired *t*-test (ΔAbs @640 nm 0.54 ± 0.01 before versus 0.48 ± 0.01 after injection; *P* = 0.004; n = 22). However, this is small in comparison with differences reported elsewhere in this paper.

**Table 3.** Effect of tamoxifen administration on cuticle deposition, pigment deposition, and egg weight (n = 22).

	Control	Tamoxifen	*P-value*
Cuticle ΔΔAbs@640 nm	–0.059 ± 0.018	0.003 ± 0.018	0.046
ΔAbs @640 nm (Δ pigment)	0.008 ± 0.008	–0.021 ± 0.009	0.058
Egg weight Δ (g)	–1.09 ± 0.36	–0.05 ± 0.49	0.053

### Experiment 3: administration of arginine vasotocin and indomethacin to influence oviposition time

As expected, the median time of oviposition after AVT injection was short, 30 min, compared to the median time of oviposition after control injections of 468 min. Injecting indomethacin prior to AVT injection blocked the advancement to some extent, the median time of oviposition after AVT injection being 168 min, although this was very variable. Injecting indomethacin alone had no effect on the median time of oviposition. The median time of oviposition after a control injection, with a prior injection of indomethacin, was 480 min compared to the median time of oviposition after control injections alone of 468 min. The predicted time of oviposition on the 28-h cycle, had no injections taken place, was 8 h after dusk and, therefore, at 450 min after the injection time. The eggs produced after AVT injections were approximately 21 h after their ovulation and 7 h prior to their expected oviposition.

**Figure 1.**
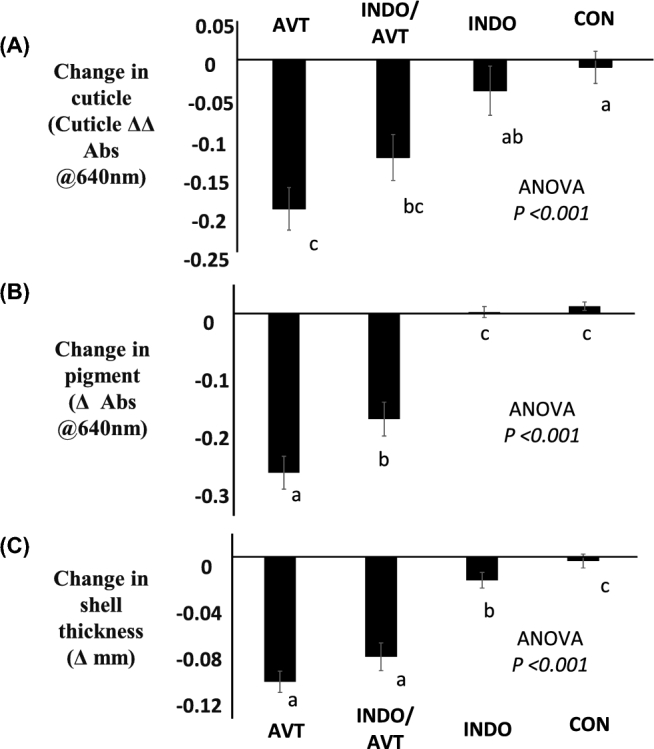
The effect of treatment with 1.05 × 10^−6^ mol/kg of AVT i.v. 7 h (AVT), 3.6 × 10^−3^ mol/kg indomethacin i.m. 10 h (INDO), their combination (INDO/AVT) or control injections (CON) prior to the time of an expected oviposition on (A) cuticle deposition, (B) pigment, and (C) shell thickness of eggs. All hens received each treatment, and the data presented are mean ± s.e.m. and were analyzed as a Latin square design ANOVA (n = 24). The experiment was carried out with four batches of six hens.

Eggs prematurely oviposited after the injection of hens with AVT alone, or in combination with indomethacin, had a significant reduction in cuticle deposition (Figure 1A), pigment (Figure 1B), and shell thickness (Figure 1C), when compared with eggs from the control hens. Indomethacin on its own had no apparent effect on the cuticle or pigment, and only a minor effect on shell thickness.

### Experiment 4: administration of arginine vasotocin to induce premature oviposition at different times in advance of a predicted oviposition

**Figure 2.**
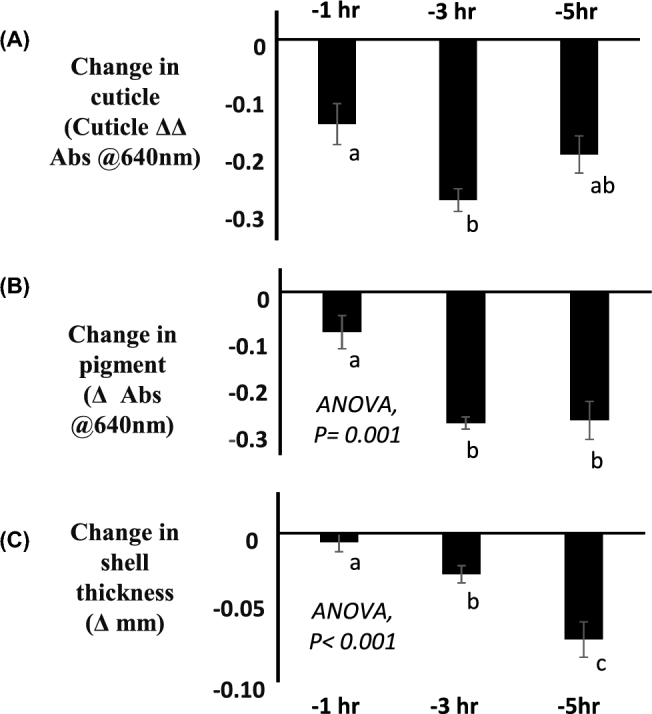
The effect of the timing (1, 3, or 5 h) of a 1.05 × 10^−6^ mol/kg of AVT i.v. injection prior to the time of an expected oviposition on (A) cuticle deposition, (B) pigment, and (C) shell thickness of eggs. All hens received each treatment, and the data presented are mean ± s.e.m. and were analyzed as a Latin square design ANOVA (n = 12). The experiment was carried out using three rooms of four hens. Columns with different letters are different at *P* < 0.05 using least significant difference.

In experiment 3, AVT was administered 7 h prior to the expected natural oviposition time. In this experiment, AVT was administered progressively closer to the expected time of oviposition, to understand when cuticle and pigment are deposited on the egg. As shown in Figure 2A, injections as close as 1 h prior (–1 h) to the expected oviposition time resulted in a level of reduction in cuticle deposition that was indistinguishable from that observed after an injection 5 h prior (–5 h) to the expected oviposition; however, the change was less than at 3 h prior (–3 h) to oviposition. When the cuticle deposition after the injection of AVT at –1 h was compared with that of a normal egg oviposited the day before treatment, rather than the effect of treatment, using a paired “t” test, it was found to be significantly less (0.10 ± 0.03 and 0.25 ± 0.01, respectively, *P* = 0.003). In the case of the deposition of pigment (Figure 2B), although there is a reduction of pigment at -1 h, this is much smaller and significantly different from the reduction observed after AVT injection at –3 or –5 hr. As shown in Figure 2C, the shell is progressively thinner, the earlier the injection was administered before the expected oviposition time. However, there was no observable difference in thickness between an egg from the same hen oviposited at –1 h, compared with a normal egg oviposited the day before treatment using a paired “t” test (0.397 ± 0.011 and 0.405 ± 0.007 mm respectively, *P* = 0.34).

### Experiment 5: administration of GNRH1 or arginine vasotocin to induce premature oviposition

**Figure 3.**
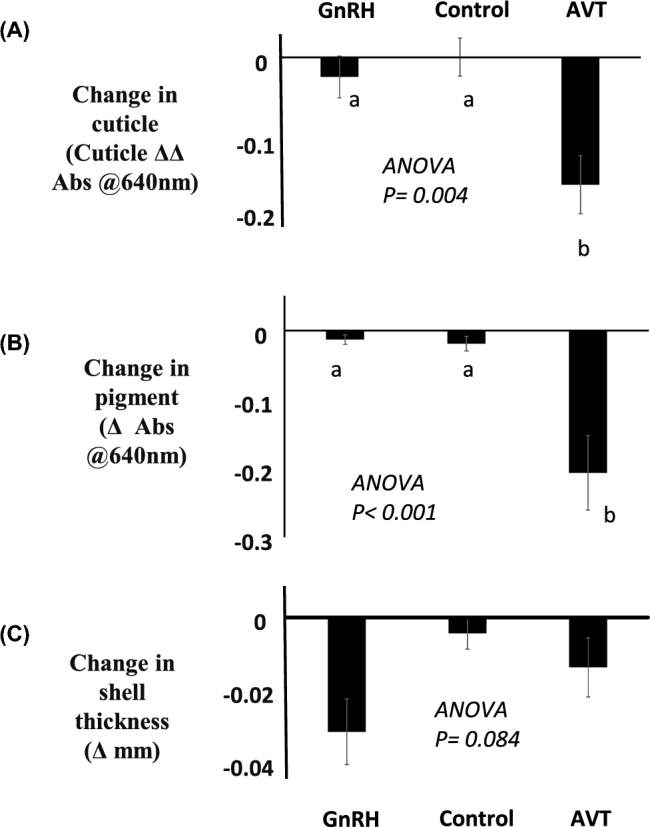
The effect of inducing a premature oviposition using 28.9 × 10^−6^ mol/kg GNRH1 i.v.(GNRH1) or 1.05 × 10^−6^ mol/kg AVT i.v. injection (AVT) or a control injection (Control) on (A) cuticle deposition, (B) pigment, and (C) shell thickness of eggs. The treated hen's eggs were laid 4–4.5 h prior to the time of an expected oviposition. All hens received each treatment, and the data presented are mean ± s.e.m. and were analyzed as a Latin square design ANOVA (n = 12). The experiment was carried out using three rooms of four hens. Columns with different letters are different at *P* < 0.05 using least significant difference.

The median time-of-lay in the control group was as predicted, while the AVT- or GNRH1- injected hens oviposited 4.5 or 4 h, respectively, prior to the predicted oviposition time. With the exception of the thickness of the shell, which was reduced (Figure 3C), the deposition of cuticle and the deposition of pigment was the same in eggs prematurely oviposited by GNRH1 injection, when compared to the control injection (Figure 3A and B). In contrast, the injection of AVT, which resulted in a premature oviposition in the same time period as the GNRH1-induced oviposition, resulted in significantly reduced cuticle and pigment deposition in comparison to the control or GNRH1 groups (Figure 3A and B). The analysis of variance did not suggest that the effect on shell thickness differed significantly across treatments; although there is a decrease in thickness with the GNRH1-induced oviposition, this is relatively modest compared to other experiments.

### Experiment 6: the effect of administration of prostaglandin on cuticle deposition

In this experiment, we directly tested if prostaglandins, which lie downstream of AVT, are involved in cuticle deposition, by comparing their effect with that of AVT. Prostaglandin had the same effect on cuticle deposition as AVT, producing a similar reduction in absorbance (Cuticle ΔΔAbs @640 nm, = –0.17 ± 0.04 versus –0.12 ± 0.05, *P* = 0.53), which was also true for the deposition of pigment (ΔAbs @640 nm (Δ pigment) = –0.08 ± 0.02 versus –0.08 ± 0.02, *P* = 0.615) and shell thickness (Δ shell thickness (mm) = –0.027 ± 0.012 versus –0.008 ± 0.005, *P* = 0.4).

### Experiment 7: the effect of the depigmentation of eggs by nicarbazin on cuticle deposition

**Figure 4.**
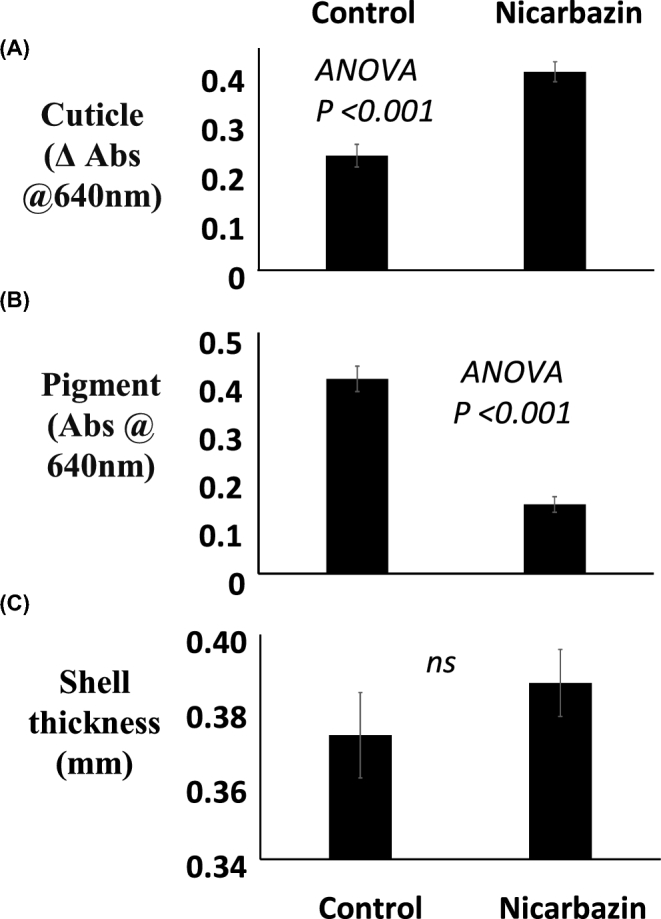
The effect of treating hens with or without 2.35 × 10^−4^ mol/kg nicarbazin in the feed on (A) cuticle deposition, (B) pigment, and (C) shell thickness of eggs. The data shown represent the direct measurement of each trait. All hens received each treatment, and the data presented are mean ± s.e.m. and were analyzed as a Latin square design ANOVA (n = 12). The experiment was carried out using two rooms of six hens.

The addition of nicarbazin to the diet induced a relatively rapid loss of pigment. Seven days after administration, the Abs @640 nm (pigment) value had reduced by over 60%, from 0.40 to 0.14 (Figure 4B). This was accompanied by ∼40% increase in the amount of cuticle measured on the same eggs (Figure 4A). As expected, there was no effect on shell thickness (Figure 4C).

### Experiment 8: the effect of a pause day on cuticle deposition

There was no difference between the pigment or cuticle deposition of eggs oviposited on a pause day, when an ovulation was missed, and those eggs oviposited the preceding day, when an ovulation occurred, when tested using a paired “t” test (before a pause day Abs @640 nm (pigment) = 0.29 ± 0.01 versus 0.28 ± 0.01 on a pause day, *P* = 0.28; Cuticle ΔAbs @640 nm before a pause day, 0.31 ± 0.02 versus 0.29 ± 0.02 on a pause day, *P* = 0.47).

### Experiment 9: immunohistochemistry on shell gland pouch and vagina tissues, after premature oviposition induced by the administration of GNRH1 or arginine vasotocin

**Figure 5.**
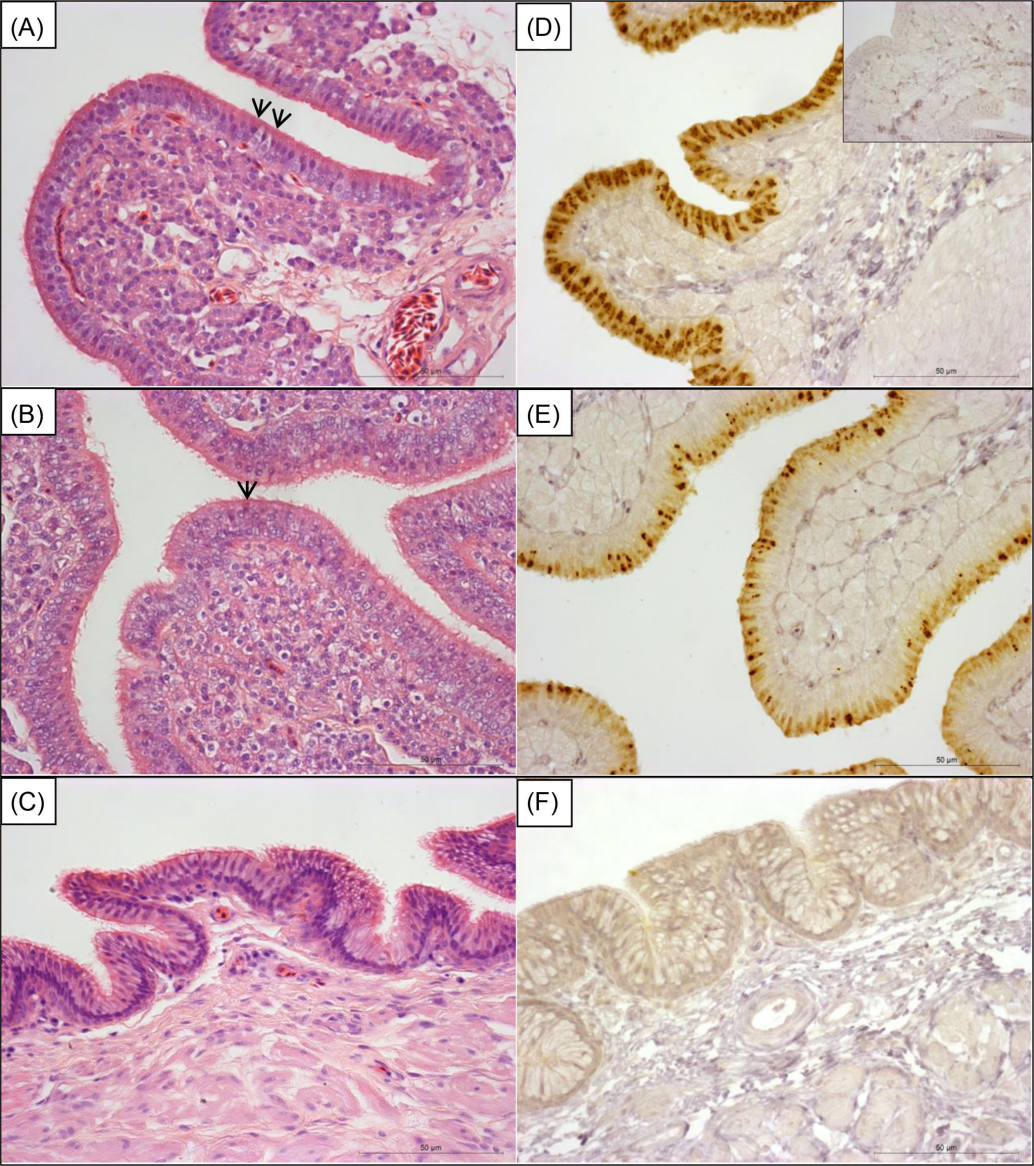
Representative images of H&E (A and B) and RARRES1 immunohistochemical staining (D and E), of the SGP from hens sampled after a premature oviposition was induced by injection of 28.9 × 10^−6^ mol/kg GNRH1 i.v. (Band E) or 1.05 × 10^−6^ mol/kg AVT i.v. (A and D). H&E (C) and RARRES1 immunohistochemical staining (F) of vagina from hens where a premature oviposition was induced by injection of AVT. The inset in panel (D) is the SGP of an AVT-injected hen where the primary antibody has been omitted (negative control). Red-staining granules (arrows) were observed in the ciliated epithelia cells of the SGP of AVT-injected hens (A). These were less abundant in the ciliated epithelial cells of the SGP of GNRH1-injected hens (B) and were absent in the epithelium lining the vagina. RARRES1 positive staining was more abundant in the ciliated epithelia cells of the SGP of AVT-injected hens (D) and less abundant in the ciliated epithelial cells of the SGP of GNRH1-injected hens (E) and were absent in the epithelium lining the vagina (F). Scale bar = 50 μm

With hematoxylin and eosin (H&E), red-staining granules were observed in the ciliated epithelia cells of the SGP of AVT-injected bird (arrows in Figure 5A). The red-staining granules were less abundant in the ciliated epithelial cells of the SGP of GNRH1-injected birds (Figure 5B) and were absent in the epithelium lining the vagina in both the AVT- (Figure 5C) and GNRH1-injected birds. Abundant positive staining was observed for RARRES1 immunohistochemistry in the ciliated epithelial cells of the SGP of AVT-injected birds (Figure 5D). Relatively sparse positive staining for RARRES1 was observed in the SGP of GNRH1-injected birds (Figure 5E). Quantification using ImageJ indicated that the % area of epithelium containing RARRES1 positive-staining granules was significantly greater in the SGP from hens after a premature oviposition had been induced by AVT, where no cuticle was deposited, than in tissues from hens where a premature oviposition had been induced by GNRH1, where a normal cuticle was deposited (22.7 ± 1.7% versus 16.0 ± 1.8%; *P* = 0.016, n = 10/11). There was no specific staining for RARRES1 in the vaginal tissue derived from AVT-injected (Figure 5F) or GNRH1-injected birds. There was no staining in the uterus of AVT-injected birds where the primary antibody was omitted (inset in Figure 5C).

## Discussion

We have demonstrated for the first time that the cuticle on an egg is susceptible to the effects of a mild environmental stressor which we know causes temporary inhibition of the reproductive axis and an increase in circulating corticosteroids [50]. However, the attempt to transiently reduce estrogenic tone by pharmaceutical means appeared to very slightly improve cuticle deposition in comparison with the control. This might be due to the partial agonist activity of tamoxifen [55], although its effect in chicks at the dose we used was reported as a pure antagonist [56]. We have demonstrated that the normal endocrine events, which are required for the ovulation of an ovum and its ultimate oviposition as a shelled egg, are necessary for the deposition of the cuticle, even if that egg is oviposited prematurely. However, causing premature oviposition using either AVT or PGF2α, which mimics only the final steps of the endocrine and paracrine events that result in oviposition, results in the absence of cuticle. Even if AVT is administered very close to the expected time of oviposition, the evidence for the deposition of cuticle is slight. This leads to two inferences: firstly, that deposition of cuticle on the egg occurs very close to the time of oviposition; and, secondly, that the cuticle is not contiguous with the organic matrix of the eggshell, but is a specific secretion which is spatially and temporally distinct from other events in the eggshell formation. While we have not ruled out a role for progesterone in cuticle deposition, it would appear that the existence of a preovulatory surge of progesterone, while an egg is in the shell gland, has no effect on cuticle deposition or pigmentation, as witnessed by the similarity between eggs from the same hen that experienced both events. We have shown that although pigment deposition and cuticle deposition are temporally close, the pigment is deposited earlier, since it is almost complete an hour before the expected oviposition, which is not the case for the cuticle.

There exists some confusion in sources of information as to where the cuticle is formed. We can say with some confidence that the cuticle is deposited in the shell gland and not the vagina. The absence of granules that stain positive for RARRES1, one of the most abundant cuticle proteins [2, 18, 54] in the vaginal epithelium of hens that laid eggs with, or without, cuticle supports the conclusion that the cuticle is not deposited in the vagina. This agrees with Romankewitsch's observation [6] that eggs recovered from the shell gland just before oviposition had cuticle coverage. So, although the vagina produces antimicrobial secretions [25, 26], these are likely to be protective of the reproductive tract rather than the egg.

The observation that the cuticle is susceptible to the effect of a mild stressor has not been reported previously to our knowledge. It is known that stress around the time of oviposition can cause egg retention and surface deposition of calcium on eggs, so an effect on the cuticle may be have a similar etiology [57, 58]. The impetus to find markers of stress that are noninvasive is a recurring topic in welfare research and has included measurements of hormones, typically in feces or eggs, and measurements of egg abnormalities or color, which has had some success [59, 60], but often the correlation is not perfect [61]. It is known that injections of adrenaline cause changes in the eggs subsequently oviposited [62], so the possibility that cuticle deposition is a sensitive integrated indicator of environmental stress warrants future examination.

It is clear that a premature oviposition induced with AVT or PGF2α results in an egg that lacks a cuticle, whereas if evoked by GNRH1 to be oviposited at the same time, the cuticle is normal. Therefore, it is not a matter of time *per se* after ovulation*.* There must instead be an event which is evoked by GNRH1, and which is not evoked by prostaglandin or AVT, that one would conclude was responsible for the deposition of the cuticle. It can also be concluded that AVT or PGF2α can be ruled out as factors that stimulate the deposition of the cuticle, despite their credentials in stimulating the muscular events leading to oviposition [45]. The most obvious factor that we have not fully tested in this series of experiments is progesterone, which has been postulated as a factor controlling cuticle deposition [38]. Experiment 8, investigating the pause-day hypothesis, indicated that eggs formed in the presence or absence of an ovulatory progesterone surge were identical for pigment and cuticle deposition. In many respects, it would make no evolutionary sense for eggs to be dependent on the ovulatory surge of progesterone for their protection. If this were the case, the final egg in any clutch would lack protection. The postovulatory follicle (POF) in chickens does contain significant amounts of progesterone [63] and probably represents an important source of circulating progesterone at the end of a clutch. It is conceivable that the duration of progesterone secretion or some other factor, as yet unidentified, from the POF is important. The simple presence of the POF is unlikely to be sufficient, since the outcomes of GNRH1 and AVT-induction are different. A signal associated with the normal endocrine cascade of ovulation and oviposition seems likely to be the key, but it does not appear to be related to the final action of AVT or prostaglandin. A possible hypothesis for the deposition of the cuticle is that a combination of the action of substances from the POF, combined with factors that act at the time of oviposition, interacts to induce deposition of the cuticle. Potential proximal factors may be the products of the sympathetic [64, 65] and parasympathetic nervous system [66]. The uterus is well innervated by these systems, [67] and they do have a role in oviposition.

It is clear that the cuticle is deposited just prior to oviposition. We have demonstrated that the termination of shell formation occurs before the deposition of the cuticle, which strongly suggests that, despite having similar constituents to the organic matrix of the shell [2], cuticle deposition is not simply an extension of shell production, but appears to be a specific event. There is also the question of the relationship between cuticle and pigment. It has been reported that the majority of the pigment is located in the outer calcified layers, with only 13–20% found in the cuticle [31], although it is commonly stated that the pigment is associated with the cuticle [30]. In the present studies, we find no direct correlation between deposition of pigment and cuticle, indeed there seems to be an increase in the amount of cuticle when the deposition of pigment is inhibited. Thus, as indicated by our previous genetic studies [2], there is no strict dependency of one event on the other. That is not to say there is no pigment in the cuticle, but rather there is no connection between the genes controlling each trait, and that the deposition of pigment is not dependent on the presence of an intact cuticle.

In conclusion, the cuticle is clearly deposited in the SGP (uterus) and not the vagina. It is susceptible to environmental stressors and requires the normal endocrine cascade leading to oviposition for deposition. However, premature oviposition induced by AVT, one of the final endocrine events prior to oviposition, even close to the expected time of oviposition does not result in the deposition of cuticle. This means the deposition of cuticle is a specific event and occurs just prior to oviposition. The cuticle is, therefore, not related directly to the organic matrix of the eggshell. The cuticle deposition is distinct from other events in eggshell formation, and although it may overlap with pigment deposition, it is not directly related to it. We have eliminated factors that might induce the deposition of the cuticle, but more work is needed to determine what does induce its release. We have made, however, significant progress in understanding basic facts about its elaboration; this will give a sound basis for future studies and the development of measures to ensure that its expression can be maximized, to reduce vertical transmission of microorganisms and contamination of eggs.

## Supplementary data

Supplementary data are available at *BIOLRE* online.

**Supplementary Table 1.** Antibodies used in the study.

Supplementary data Supplementary data are available at *BIOLRE* online.
